# Early Outcomes of MDR-TB Treatment in a High HIV-Prevalence Setting in Southern Africa

**DOI:** 10.1371/journal.pone.0007186

**Published:** 2009-09-25

**Authors:** Kwonjune J. Seung, David B. Omatayo, Salmaan Keshavjee, Jennifer J. Furin, Paul E. Farmer, Hind Satti

**Affiliations:** 1 Division of Global Health Equity, Brigham and Women's Hospital, Boston, Massachusetts, United States of America; 2 Partners In Health, Lesotho, Maseru, Lesotho; McGill University, Canada

## Abstract

**Background:**

Little is known about treatment of multidrug-resistant tuberculosis (MDR-TB) in high HIV-prevalence settings such as sub-Saharan Africa.

**Methodology/Principal Findings:**

We did a retrospective analysis of early outcomes of the first cohort of patients registered in the Lesotho national MDR-TB program between July 21, 2007 and April 21, 2008. Seventy-six patients were included for analysis. Patient follow-up ended when an outcome was recorded, or on October 21, 2008 for those still on treatment. Fifty-six patients (74%) were infected with HIV; the median CD4 cell count was 184 cells/μl (range 5–824 cells/μl). By the end of the follow-up period, study patients had been followed for a median of 252 days (range 12–451 days). Twenty-two patients (29%) had died, and 52 patients (68%) were alive and in treatment. In patients who did not die, culture conversion was documented in 52/54 patients (96%). One patient had defaulted, and one patient had transferred out. Death occurred after a median of 66 days in treatment (range 12–374 days).

**Conclusions/Significance:**

In a region where clinicians and program managers are increasingly confronted by drug-resistant tuberculosis, this report provides sobering evidence of the difficulty of MDR-TB treatment in high HIV-prevalence settings. In Lesotho, an innovative community-based treatment model that involved social and nutritional support, twice-daily directly observed treatment and early empiric use of second-line TB drugs was successful in reducing mortality of MDR-TB patients. Further research is urgently needed to improve MDR-TB treatment outcomes in high HIV-prevalence settings.

## Introduction

Multidrug-resistant tuberculosis (MDR-TB), defined as resistance to both isoniazid and rifampicin, is a growing problem in resource-poor settings where adequate diagnosis and treatment are often unavailable. Sub-Saharan Africa would seem to have all of the conditions for a “perfect storm” of HIV infection and MDR-TB [1]. In the most recent World Health Organization (WHO) report on anti-tuberculosis drug resistance, data were noticeably absent from African countries, which often do not have the resources to perform regular drug resistance surveillance [2]. Nevertheless, there is ample evidence that MDR-TB is a major problem in Africa. In 2006, extensively drug-resistant tuberculosis (XDR-TB), defined as MDR plus resistance to fluoroquinolones and second-line injectable drugs, was found in South Africa. It spread rapidly among HIV immunocompromised patients and was associated with extremely high mortality [3]. The WHO reported that, even in the absence of a nationwide survey, South Africa identified 17,615 MDR-TB isolates over a 4-year period, 996 (5.6%) of which were XDR-TB [2].

Treatment of MDR- and XDR-TB is difficult even in resource-rich settings. Patients are generally treated for a minimum of 18–24 months with second-line TB drugs that have significant adverse effects [4]. Most studies of MDR-TB treatment have come from countries where HIV co-infection is uncommon [5], [6]. Thus, little is known about outcomes of MDR-TB treatment in high HIV-prevalence settings such as sub-Saharan Africa.

Lesotho is a mountainous nation that is home to two million people and completely surrounded by the Republic of South Africa. With respect to HIV and TB, Lesotho is very similar to its neighbor, South Africa, whereas its medical infrastructure and resources reflect its significantly lower per capita GDP. It has one of the highest burdens of HIV infection in the world, with a prevalence of 23.2% [7]. The prevalence of TB is estimated to be 513 cases per 100,000 population [8]. An estimated 80% of all TB patients are co-infected with HIV [9]. Accurate data on drug resistance are currently unavailable, pending the results of a nationwide drug resistance survey that was started in 2008.

The Lesotho Ministry of Health and Social Welfare recently began a national treatment program as part of a comprehensive response to MDR- and XDR-TB. International collaborators included Partners In Health, the Open Society Institute, the Foundation for Innovative New Diagnostics (FIND), and the World Health Organization. Through this program, quality-assured second-line TB drugs procured through the Green Light Committee Initiative (GLC) [10] were available to patients diagnosed with MDR-TB.

We conducted a retrospective analysis of early outcomes of MDR-TB treatment in the first cohort of patients treated in this program. To our knowledge, this is the first analysis of MDR-TB treatment outcomes in the midst of a generalized HIV epidemic in a resource-poor setting. It is also the first report of a community-based MDR-TB treatment model in sub-Saharan Africa.

## Methods

We retrospectively examined the medical records of consecutive patients registered by the Lesotho national MDR-TB program between July 21, 2007 and April 21, 2008. This included all patients starting treatment with second-line TB drugs for confirmed or presumptive MDR-TB.

### Ethics

As this was a retrospective study of medical information previously collected in the course of routine clinical care, informed consent was not required. This study was approved by the Human Subjects Committee of the Harvard School of Public Health.

### Bacteriology and drug susceptibility testing

At the beginning of the study period, the Lesotho National Reference TB Laboratory performed direct smear microscopy without fluorescence, but not culture or drug susceptibility testing (DST). Some patients brought DST results from various public and private sector TB laboratories in South Africa showing resistance to isoniazid and rifampicin. These patients were immediately started on treatment with second-line TB drugs, and were asked to produce sputum for culture and DST before starting treatment. Sputum was sent to the Medical Research Council (MRC) Tuberculosis Laboratory, a supranational reference laboratory in Pretoria, South Africa for first- and second-line DST.

Starting in 2007, the Lesotho National Reference TB Laboratory began receiving training from FIND in culture and DST. Culture was performed initially on Löwenstein-Jensen medium, and later using a BACTEC MGIT 960 system (Becton-Dickson, Sparks, Maryland, USA). DST was performed to the first-line drugs of isoniazid, rifampicin, ethambutol and streptomycin. MDR isolates continued to be sent to MRC for DST to pyrazinamide and second-line drugs throughout the entire study period.

### Treatment

In general, patients were referred to the Lesotho national MDR-TB program for evaluation of confirmed or suspected MDR-TB. Most patients were referred by clinicians working in the Ministry of Health and Social Welfare network of hospitals and health centers, but some were referred by private physicians or South African mining companies. Patients with suspected MDR-TB but without DST results were categorized according to clinical and bacteriological criteria, and high-risk patients were started empirically on second-line TB drugs. High-risk categories included: household contacts of known MDR-TB patients; probable treatment failure of a WHO Category 1 or Category 2 regimen (defined as smear-positive in month 5, or HIV-positive and clinical deterioration at any point during treatment); and history of treatment with second-line TB drugs (full protocol shown in Table 1).

**Table 1:** Protocol for empiric treatment of MDR-TB suspects.

Risk level	Category	Action
Medium risk	Migrant worker with new TB	Send two sputums for culture and DST. Start Category 1 regimen.
Medium risk	Health worker with new TB	Send two sputums for culture and DST. Start Category 1 regimen.
Medium risk	Treatment after relapse or default	Send two sputums for culture and DST. Start Category 2 regimen.
High risk	Household contact of known MDR-TB patient with new TB	Send two sputums for culture and DST. Start individualized Category 4 regimen based on DST of contact.
High risk	Probable treatment failure:• Smear-positive in fifth month of Category 1 or 2, or• HIV-positive and clinically worsening during Category 1 or 2	Send two sputums for culture and DST. Start standardized Category 4 regimen. There are many reasons for clinical worsening in HIV-positive patients besides treatment failure. Consult specialist for advice.
High risk	History of treatment with second-line drugs	Send two sputums for culture and DST. Will need an individualized Category 4 regimen. Consult specialist for advice.

Treatment protocols followed WHO international guidelines [11]. A standardized treatment regimen was used for empiric treatment in patients who had previously received only first-line TB drugs. The standardized regimen included six drugs—pyrazinamide, kanamycin, ofloxacin, ethionamide, cycloserine and para-aminosalicylic acid. In patients who had previously taken second-line TB drugs, empiric treatment regimens were designed wherever possible to avoid drugs that had been taken previously. When second-line DST results were available, the treatment regimens were modified accordingly, with the goal of at least five drugs to which isolates were susceptible. All TB drugs were dosed according to weight. Dosing and drugs were also changed in response to severe adverse effects. In order to monitor treatment, sputum samples were taken monthly for smear microscopy and culture.

HIV testing was routinely offered at the first consultation to patients who did not know their HIV serostatus. HIV-positive patients who were not already taking antiretroviral therapy (ART) were started on ART as soon as they were tolerating second-line TB drugs, irrespective of CD4 cell count. ART regimens commonly consisted of two nucleoside reverse transcriptase inhibitors (zidovudine or stavudine plus lamivudine), and one non-nucleoside reverse transcriptase inhibitor (efavirenz or nevirapine).

Patients in stable clinical condition were started on MDR-TB treatment on an ambulatory basis. Patients were evaluated at least monthly by a trained clinician. All doses of second-line TB drugs and ARV's were administered under twice-daily directly observed treatment (DOT). Community health workers were trained to provide DOT, monitor for adverse effects and provide psychosocial support. Patients received daily injections from health centre nurses or by community health workers if living in remote areas. Patients who were unable to ambulate or were otherwise unstable clinically were admitted to a specialized 18-bed MDR-TB inpatient facility in the capital (Botsabelo Hospital). Patients were also admitted if they experienced severe adverse effects or other clinical complications that could not be managed as outpatients. All treatment was free of charge. Patients received a monthly food package and were reimbursed for treatment-related travel expenses. Community health workers were reimbursed for travel expenses and were provided with a monthly cash incentive.

### Data collection and analysis

Variables collected through a standardized chart abstraction included: demographic characteristics; previous TB treatment exposure; previous exposure to antiretroviral therapy; HIV serostatus; cavitary and bilateral disease on chest radiography; results of drug susceptibility testing; results of hemoglobin, creatinine, and hepatic function tests at baseline and during treatment, and occurrence of serious adverse effects and clinical complications (definitions shown in Table 2).

**Table 2:** Definition of serious adverse effects and clinical complications during MDR-TB treatment.

Pneumothorax	Pneumothorax visible on chest radiograph and requiring chest tube placement
Hematemesis	Episode seen and documented by any health care worker
Otoxicity	Symptomatic hearing loss while receiving injectable drug
Renal insufficiency	Elevated creatinine requiring suspension or dose reduction of injectable drug
Severe nausea and vomiting	Nausea and vomiting requiring intravenous fluid repletion
Depression	Depression treated with anti-depressants and documented by a physician
Seizures	Episode of seizures documented by a physician
Psychosis	Symptoms treated with anti-psychotics and documented in the clinical record to be due to cycloserine by a physician
Neuropathy	Chronic tingling, numbness or pain in the extremities requiring change in ART or TB treatment regimen or treatment with amitriptyline
Severe anemia	Hemoglobin <8 g/dL
Hypokalemia	Serum potassium <3.0 mmol/L

Patient follow-up ended when an outcome was recorded, or on October 21, 2008 for those still on treatment. Possible outcomes included: death; default (greater than one month of missed doses); transfer out (transfer with records to another MDR-TB treatment program); and alive and in treatment. Autopsy was not available; cause of death was determined after a detailed review of clinical records and was a consensus decision of the first and last authors. Univariate analyses (χ^2^ test) were performed to determine the associations between clinical characteristics and outcome. Multiple logistic regression analysis was performed to look for potential confounders or effect modifiers. SAS software (version 9.1, Cary Institute, North Carolina, USA) was used for all analysis.

## Results

Between July 21, 2007 and April 21, 2008, 81 patients started treatment with second-line TB drugs for presumptive MDR-TB. Three patients were subsequently found to be infected with pan-susceptible *M. tuberculosis* strains, and two patients were subsequently found to be infected with polyresistant, non-MDR strains. These five patients were excluded from analysis. Baseline characteristics of the remaining 76 patients are shown in Table 3.

**Table 3:** Baseline characteristics of MDR-TB patients in Lesotho.

	N (%)	Median (range)
**Demographics**		
Female	36 (47.4%)	
Age		35.0 (3 – 62)
Months since first diagnosis of TB		16.9 (0 – 316.7)
**Clinical**		
HIV seropositive	56 (73.7%)	
Body mass index		17.5 (11.9 – 26.8)
Serum albumin (g/L)		31.3 (13 – 48)
Hemoglobin (g/dL)		11.3 (6.1 – 16.5)
Bilateral disease on CXR†	32 (46.4%)	
Cavitary disease on CXR†	33 (47.8%)	
**Previous treatment**		
Number of previous treatment regimens		2 (0 – 5)
Treatment with second-line drugs	6 (15%)	
Previous treatment in South Africa	8 (20%)	
**Household contacts**		
Number of household contacts		4.0 (0 – 11)

† Out of 69 patients who had a CXR dated within 90 days before or after the initiation date of second-line TB drugs.

Seventy patients (92%) were sputum AFB-positive before starting treatment. Of the remainder, five patients were smear- and culture-negative, including three children; the final patient was smear-negative but culture-positive. Twenty-nine patients (38%) were started on treatment with second-line TB drugs already having laboratory confirmation of resistance to isoniazid and rifampicin, mostly from South African laboratories. Forty-seven patients (62%) were started on empiric treatment with second-line TB drugs without having laboratory-confirmed MDR-TB. All patients starting empiric MDR-TB treatment had sputum samples sent for culture and DST, and 26 of these patients eventually proved to have MDR-TB; the remaining 21 patients had negative or contaminated cultures. Ten MDR isolates were tested by MRC in South Africa for resistance to second-line drugs. Of these, four isolates were found to be resistant to second-line drugs; one isolate met criteria for XDR-TB.

Only two patients had never been treated for TB. Seventy-four patients (97%) had previously received TB treatment, ranging from one to five previous courses of TB treatment (Table 4).Usually this was with repeated treatments with first-line drugs, but 12 patients (16%) reported a history of treatment with second-line TB drugs, usually in South Africa.

**Table 4:** Previous TB treatment history of MDR-TB patients in Lesotho.

Number of previous TB treatments	N (%)
Zero	2 (3%)
One	24 (32%)
Two	35 (46%)
Three	11 (14%)
Four	3 (4%)
Five	1 (1%)

Fifty-six patients (74%) were infected with HIV; of the 32 patients who were tested within 90 days before or after initiation of MDR-TB treatment, the median CD4 cell count was 184 cells/μl (range 5–824 cells/μl). Of the HIV-positive patients, 24 patients were already receiving ART, a mean of 369 days (range 13 days to more than 3 years) before initiation of MDR-TB treatment. Twenty-five patients were started on ART, a mean of 35 days (range 11–117 days) after starting MDR-TB treatment. Of the remaining seven patients, six patients died without starting ART, and one was alive, in treatment and culture-negative at the end of the follow-up period.

**Figure 1:**
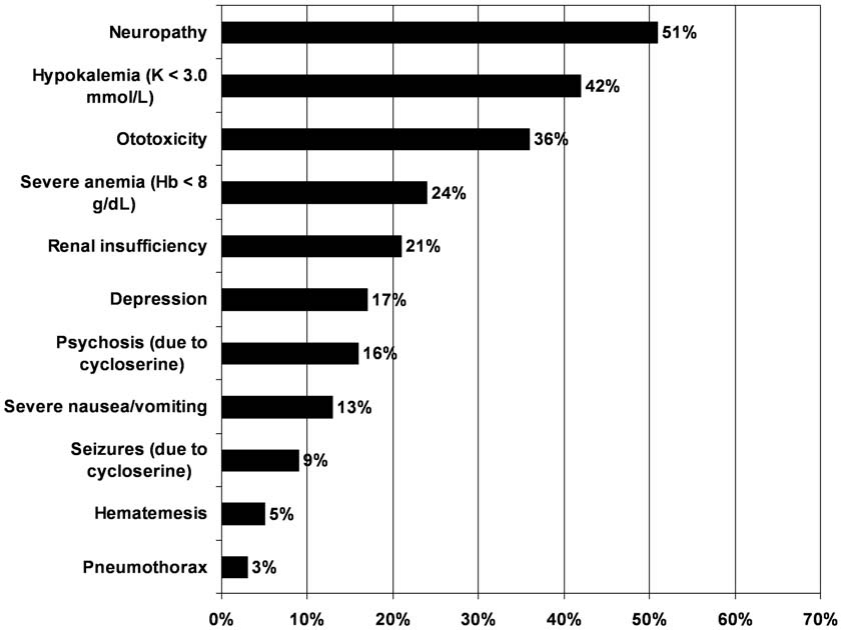
Major adverse effects and clinical complications during MDR-TB treatment. Adverse effects and clinical complications were defined as in Table 2. Percentages were calculated out of a total of 76 patients.

Fifty-eight patients (76%) experienced at least one hospitalization during the follow-up period. Hospitalization ranged from 2 to 188 days, and the mean length of stay was 33 days. Seventy patients (92%) experienced at least one serious adverse effect or clinical complication during the follow-up period (Figure 1).

**Figure 2:**
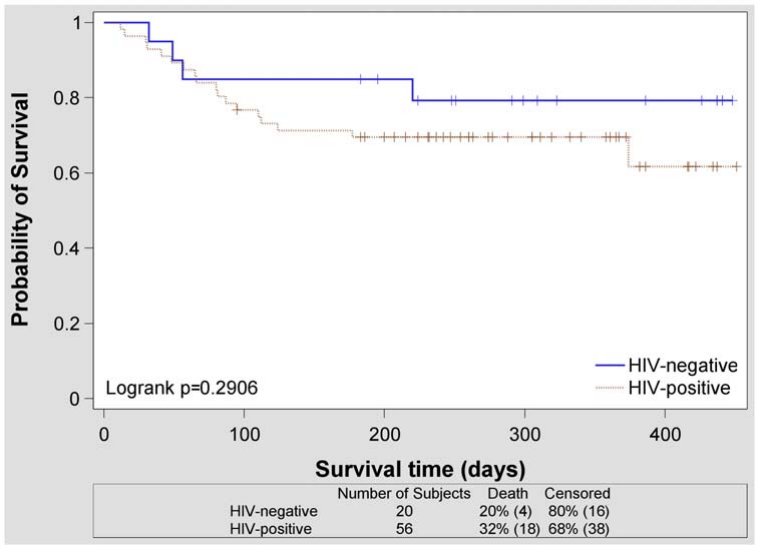
Time to death during MDR-TB treatment by HIV status. This figure shows Kaplan-Meier probabilities of survival in HIV-negative (solid line) and HIV-positive (dotted line) patients. Survival is measured in days after starting MDR-TB treatment. Crosses indicate patients who did not die by the end of the follow-up period.

By October 21, 2008, study patients had been followed for a median of 252 days (range 12–451 days). Twenty-two patients (29%) had died, and 52 patients (68%) were alive and in treatment. One patient had defaulted, and one patient had moved to South Africa after culture conversion and was continuing to receive treatment at another institution. In patients who did not die, culture conversion—defined as two consecutive negative cultures greater than 30 days apart [12]—was documented in 52/54 patients (96%). Death occurred after a median of 66 days in treatment (range 12–374 days). Survival curves stratified by HIV status are shown in Figure 2. There appeared to be a non-significant trend towards earlier death for patients who were HIV-positive (dotted line). The causes of death for the 22 patients who died are listed in Table 5.

**Table 5:** Cause of death of patients who died during MDR-TB treatment.

Cause of death	Number
Unknown	10
Multiorgan system failure (disseminated TB)	3
Massive upper gastrointestinal hemorrhage	3
Meningitis, unknown etiology	2
Progressive respiratory failure	1
Severe COPD exacerbation	1
Injectable-related renal failure	1
Pneumothorax	1
Total	22

In univariate analyses, hemoglobin <10 g/dL, serum albumin <30 g/L, and an age >40 were associated with death (Table 6). In multiple logistic regression analysis, there was a suggestion of confounding or interaction between all three of these covariates, but the sample size precluded definitive conclusions.

**Table 6:** Covariates associated with risk of death during MDR-TB treatment

Covariates	OR	95% CI	p-value (χ^2^)
Male gender	2.5	0.9 – 7.1	0.08
HIV seropositive	1.9	0.6 – 6.5	0.30
Baseline albumin <30 g/L	3.9	1.3 – 11.6	0.01
Baseline hemoglobin <10 g/dL	3.3	1.2 – 9.4	0.02
Cavitary disease	0.5	0.2 – 1.3	0.19
Bilateral disease	1.2	0.4 – 3.3	0.80
Age >40	2.9	1.0 – 8.0	0.04
BMI <18.4	2.2	0.8 – 6.3	0.09
Number of previous treatments > = 3	1.9	0.6 – 6.1	0.29
Laboratory-confirmed MDR-TB	1.0	0.3 – 3.1	0.96

## Discussion

While there have been many previous studies of MDR-TB treatment outcomes in resource-poor settings, none have included significant numbers of HIV-positive patients [13]. In MDR-TB treatment programs with largely HIV-negative patients, cure rates have ranged from 61–77%, and death rates have ranged from 5–19% [5], [6], [14], [15]. The early results of treatment in Lesotho, however, indicate that MDR-TB treatment outcomes in HIV-positive patients are likely to be significantly worse compared to HIV-negative patients. There was a trend towards poorer outcomes in HIV-positive patients, with significant mortality in the first few weeks after initiation. These results are consistent with previous studies showing increased mortality in HIV-positive compared to HIV-negative patients with drug-susceptible TB [16], [17]. A high rate of HIV co-infection is one of the major reasons for the high mortality rate among drug-susceptible TB patients in sub-Saharan Africa. Harries et al. reported a 23% death rate among a large cohort of TB patients in Malawi [18]. Alvarez et al. reported a 26.5% death rate among patients with confirmed pulmonary TB admitted to a South African hospital, with a median time of death of 25 days [19].

The high early mortality seen in this cohort argues strongly for early initiation of second-line TB drugs in MDR-TB patients. One common theme in prior studies of HIV-positive patients with MDR-TB is the rapidity of death in the absence of effective treatment. Several studies in the 1980s and 1990s of MDR-TB in HIV co-infected patients from Europe and the United States reported mortality rates exceeding 70%, often within 4–8 weeks of diagnosis, and before laboratory confirmation of MDR-TB [1]. These reports were from the pre-ART era, not that different from today's situation in many parts of Africa, where access to ART is still limited. In a 2006 report from the KwaZulu-Natal province of South Africa, 52/53 (98%) of patients with XDR-TB died a median of 16 days from the time of diagnosis [3].

Clinicians are often reluctant to start second-line TB drugs empirically, without laboratory confirmation of resistance to isoniazid and rifampicin. But in situations where DST is not easily accessible, the reliance on laboratory diagnosis can become a bottleneck to life-saving treatment. In this cohort of patients, most patients were started empirically on second-line drugs, without waiting for DST results. Almost all of these patients were sputum smear-positive and would be expected to have positive cultures. However, for many patients, the cultures ended up being negative or contaminated, since in the initial phase of the program, the raw sputum had to be transported over long distances to a laboratory in South Africa. Even in settings where there is ready access to culture and DST, empiric MDR-TB treatment is a strategy that can decrease the time to effective treatment. The most commonly available DST methods utilize culture on solid or liquid media, which, at a minimum, require several weeks or months, ample time for a patient to clinically worsen or die while receiving ineffective first-line TB drugs. With the aid of clear guidelines and protocols, clinicians can be trained to identify patients at high risk for MDR-TB and initiate second-line TB drugs empirically. There is much to be gained and little to be lost by a starting a well-designed empiric MDR-TB treatment regimen in high-risk patients [20], [21], [22]. If DST subsequently shows the patient to be infected with drug susceptible TB, the patient can be simply switched back to a first-line regimen.

In this cohort, the majority of patients (62%) received empiric MDR-TB treatment, and therefore benefited from effective therapy earlier than they would have normally. Nevertheless, by many objective measures, they did not benefit early enough. In this largely HIV-positive population with advanced immunosuppression, a high proportion of smear-negative patients would normally be expected. In fact, 92% of the patients were smear-positive. Many of them may have originally had smear-negative pulmonary TB, but by the time they were referred for suspicion of MDR-TB, the sputum bacillary load was much higher. Ninety-seven percent were retreatment cases, with 65% previously receiving two or more TB treatments. This means that for most patients, there had previously been multiple missed opportunities for MDR-TB diagnosis before referral. The most common pathway to a diagnosis of MDR-TB in resource-poor settings is through multiple failed treatments with standardized regimens of first-line TB drugs. This strategy is unlikely to result in acceptable MDR-TB treatment outcomes in high HIV-prevalence settings, even in those settings with greater medical resources than Lesotho, such as South Africa. For the first cohort of MDR-TB patients treated in Lesotho, where DST and second line TB drugs had previously been unavailable, this sort of delayed diagnosis may be understandable, but probably was one of the factors responsible for the high early mortality. In the future, earlier diagnosis of MDR-TB, and in particular, increasing the diagnosis of MDR-TB among new TB patients, as has been done in other countries with more mature MDR-TB treatment programs [6], will likely be necessary to improve overall treatment outcomes.

Another major bottleneck to MDR-TB treatment in the southern African region is a reliance on hospital-based treatment. In many countries, patients with MDR-TB are routinely admitted to the hospital for several months at the start of treatment. This is partly for clinical reasons and partly to protect the community from infection [23]. Unfortunately, if the number of MDR-TB cases outstrips the supply of hospital beds, patients may have to queue for treatment. In certain areas of South Africa, patients may wait for several months before an MDR-TB hospital bed opens up [24]. Ironically, this policy likely increases community transmission of MDR- and XDR-TB strains, since patients continue to be infectious in the community without effective treatment. Furthermore, the reliance on hospital-based MDR-TB treatment also likely increases nosocomial transmission, since MDR-TB wards in resource-poor settings often have no additional infection control measures than do general hospital wards [25].

In Lesotho, a community-based model of MDR-TB treatment was found to be feasible, as it has been in other countries [14], [26]. Community health workers were successfully trained to support patients, observe doses, provide injections, and monitor for adverse effects. All outpatients received twice-daily DOT from a trained community health worker. Only patients requiring a higher level of care were admitted to the hospital; once they were medically stable and able to care for themselves with assistance from family, they were discharged back to community-based care. This relationship between hospital-based and community-based staff requires close communication, but maximizes programme resources for the largest number of patients and provides close follow-up throughout all stages of treatment. Given the serious outbreak of MDR-TB in the southern Africa region and the limited public health response to date, improved hospital facilities are certainly needed. A strong community-based treatment programme, however, should be an important aspect of any national response to MDR-TB.

In Lesotho, the default rate appeared to be quite low compared to typical default rates of 15–20% among MDR-TB patients in South Africa [27], [28]. One advantage of community-based DOT over self-administered treatment is closer monitoring of adverse effects. Adverse effects and other clinical complications occurred early and often in Lesotho, compared to HIV-negative settings [29], [30], [31]. HIV-positive patients are known to have an increased incidence of adverse effects of first-line TB drugs [32], [33], [34], and the incidence of adverse effects of second-line TB drugs may be increased as well. Some adverse effects, such as seizures and psychosis, were most likely due to second-line TB drugs (e.g., cycloserine). Others, such as peripheral neuropathy, are known adverse effects of second-line TB drugs, but can also be caused or exacerbated by other conditions (e.g., HIV peripheral neuropathy) or other drugs (e.g., stavudine) commonly taken by HIV-positive patients. We surmise that the increased frequency and rapid onset of many side effects and clinical complications, such as hypokalemia, anemia or renal insufficiency, were related to the poor clinical status and baseline malnutrition prevalent in this patient population, but further research is needed.

Community health workers also play an important role in ensuring that patients receive the psychological, social, and economic support required to complete treatment. Community-based MDR-TB treatment should include an assessment of the home situation and nutritional intervention when necessary. In Lesotho, all patients treated for MDR-TB were provided with a monthly food package. The high prevalence of low BMI and hypoalbuminemia in our cohort was probably caused by chronic malnutrition, not just chronic TB and HIV infection. MDR-TB patients in this region likely suffer from energy, protein and micronutrient deficiencies, all of which can contribute to early mortality, side effects and clinical complications in patients with TB or HIV [35], [36], [37].

### Limitations

There are several caveats and limitations to this analysis that should be considered before extrapolating to other patient populations and programs. This analysis included patients who did not have DST. We chose to do this because our treatment protocols strongly promoted empiric treatment of MDR-TB, and DST was not readily available during the entire study period. The inclusion of non-MDR strains, furthermore, would not have affected the results reported here, since according to national protocol, the standardized MDR-TB treatment regimen did not include isoniazid or rifampicin. No patients in this cohort benefited from the superior activity of these drugs, even in the event that some patients were infected with pan-susceptible TB. The early outcomes reported here may be different from the final outcomes; reporting of MDR-TB treatment outcomes generally requires more than two years of follow-up, in order to fully account for late death, treatment failure, default, and recurrent disease [12]. The size of the cohort was too small to do meaningful multivariable analysis; univariate associations will have to be reexamined in larger cohorts. Finally, the incidences of some important adverse effects, most notably hypothyroidism, are not reported here, because they were not included in the screening protocol in the beginning of this program.

In a region where clinicians and program managers are increasingly confronted by MDR- and XDR-TB, this report provides sobering evidence of the difficulty of MDR-TB treatment in high HIV-prevalence settings. Nevertheless, in Lesotho, an innovative community-based treatment model that involved social and nutritional support, strict DOT, and early empiric use of second-line TB drugs was successful in reducing mortality of MDR-TB patients. Further research is urgently needed to improve MDR-TB treatment outcomes in high HIV-prevalence settings.
